# The order of concurrent training affects mTOR signaling but not mitochondrial biogenesis in mouse skeletal muscle

**DOI:** 10.14814/phy2.14411

**Published:** 2020-04-13

**Authors:** Takanaga Shirai, Yuki Aoki, Kohei Takeda, Tohru Takemasa

**Affiliations:** ^1^ Graduate School of Comprehensive Human Sciences University of Tsukuba Tsukuba Japan; ^2^ Faculty of Health and Sport Sciences University of Tsukuba Tsukuba Japan

**Keywords:** concurrent training, mitochondrial biogenesis, mTOR signaling, skeletal muscle

## Abstract

Concurrent training involves a combination of two different modes of training. In this study, we conducted an experiment by combining resistance and endurance training. The purpose of this study was to investigate the influence of the order of concurrent training on signal molecules in skeletal muscle. The phosphorylation levels of p70 S6 kinase, S6 ribosomal protein, and 4E‐binding protein 1, which are related to hypertrophy signaling, increased significantly in the resistance–endurance order group as compared with in control group not the endurance–resistance order group. The gene expressions related to metabolism were not changed by the order of concurrent training. The mitochondrial respiratory chain complex was evaluated by western blot. Although both groups of concurrent training showed a significant increase in MTCO1, UQCRC2, and ATP5A protein levels, we could not detect a difference based on the order of concurrent training. In conclusion, a concurrent training approach involving resistance training before endurance training on the same day is an effective way to activate both mTOR signaling and mitochondria biogenesis.

## Graphical abstract

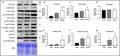

## 1 INTRODUCTION

In many sports, athletes aim to output their maximum performance during competitions or games. To consistently achieve this, improving their strength and endurance by as much as possible is crucial (Camera, Smiles, & Hawley, 2016). Skeletal muscle is one of the major tissues wherein the training effects appear most remarkably. Previous studies have elucidated the differences in training methods and found that each of them can improve either strength, endurance, or both of them. Chronic muscle contraction induces morphological and metabolic adaptations because of the cumulative effects of repeated bouts of exercise. Exercise training is mainly divided into two types. One is resistance exercises like weightlifting or powerlifting that cause skeletal muscle hypertrophy and strength improvements (Ogasawara, Kobayashi, et al., 2013; Ogasawara, Yasuda, Sakamaki, Ozaki, & Abe, 2011). The other is endurance exercises such as long‐distance running or cycling that lead to skeletal muscle oxidative and metabolic capacity increase (Fan & Evans, 2017; Holloszy & Coyle, 1984).

Concurrent training is the combination of two different modes of training, which is intended to achieve both types of effects (Hickson., 1980). However, it is also known that an interference effect may appear that weakens the outcome of performing both kinds of training during a single period (Hickson., 1980). The phenomenon of the interference effect can be demonstrated at molecular levels in skeletal muscles (Coffey & Hawley, 2017).

Resistance training induces muscle hypertrophy by activating the mammalian/mechanistic target of rapamycin (mTOR) signaling pathway, which contains p70 S6 kinase (p70S6K) and S6 ribosomal protein (S6) (Mayhew, Kim, Cross, Ferrando, & Bamman, 2009; Ogasawara, Yasuda, Ishii, & Abe, 2013). Moreover, endurance training activates adenosine monophosphate (AMP)‐activated protein kinase (AMPK) by way of AMP produced by adenylate kinase (Jaspers et al., 2017). Consequently, peroxisome proliferator‐activated receptor γ coactivator‐1α (PGC‐1α), a master regulator of energy metabolism and mitochondrial biogenesis, is activated and the metabolic capacity is improved (Olesen, Kiilerich, & Pilegaard, 2010). However, AMPK interferes with mTOR signaling via tuberous sclerosis complex 2 (TSC2) and is thought to suppress protein synthesis (Inoki, Zhu, Guan, & Arbor, 2003). According to previous research that compared concurrent exercise and resistance exercise alone, it was found that the total level of p70S6K in concurrent exercise is lower than that of resistance exercise alone (Apro, Wang, Ponten, Blomstrand, & Sahlin, 2013). The reason for this is the interference effect that occurs between resistance and endurance exercise. Another study reported that the last exercise performed in an acute concurrent exercise regimen with resistance and endurance exercise determines the ultimate molecular response (Ogasawara, Sato, Matsutani, Nakazato, & Fujita, 2014). Overall, it has been considered difficult to establish an effective combination training plan that increases both muscular strength and endurance.

However, the effects of concurrent training on muscle hypertrophy and endurance enhancing signals have not yet been clarified. Therefore, in this study, we sought to clarify the difference in the order to concurrent training on skeletal muscle, focusing on mTOR, and mitochondria biogenesis signals.

## 2 MATERIALS AND METHODS

### 2.1 Animals

All experimental procedures performed in this study were approved by the Institutional Animal Experiment Committee of the University of Tsukuba (15‐057). Male Institute of Cancer Research (ICR) mice aged 7–8 weeks old (Tokyo Laboratory Animals Science Co.) were used in this study. The mice were housed in temperature (22 ± 2°C) and humidity (55 ± 5%)‐controlled holding facilities under a 12‐/12‐hr light/dark cycle and had ad libitum access to food and water. Upon completion of experimental treatments, the mice were killed by cervical dislocation. Their lower limb muscles were then dissected 24 hr after the last exercise session, weighed quickly frozen in liquid nitrogen, and stored at −80°C until needed for analysis.

### 2.2 Training methods

#### 2.2.1 Resistance exercise protocol

The resistance exercise protocol was carried out as previously described (Ogasawara et al., 2014, 2016). Briefly, under inhaled isoflurane anesthesia (2%, KN‐1701; Natsume), the lower both legs of each mouse was shaved and cleaned with alcohol wipes.

The mice were positioned with their foot on a footplate (with an ankle joint angle of 90°) in the prone posture. The triceps calf muscle was stimulated percutaneously with electrodes connected to an electric stimulator and isolator (Ag/AgCl, Vitrode V; Nihon Kohden). The gastrocnemius muscle was isometrically exercised (three‐second stimulation, 10 contractions, with 7‐s intervals between contractions; total of five sets with 3‐min intervals between sets). The voltage (30V) and stimulation frequency (100 Hz) were adjusted to produce maximal isometric tension. Previous study demonstrated that this exercise protocol is known to increase anabolic signaling activity (Ogasawara et al., 2014) and induces significant muscle hypertrophy, simulating long‐term training (Ogasawara, Kobayashi, et al., 2013).

#### 2.2.2 Endurance exercise protocol

The endurance exercise protocol was carried out as previously described (Ogasawara et al., 2014). The mice were familiarized with running on a rodent treadmill at 10–20 m/min for 3 days prior to the experiment. Subsequently, they were placed on a flat treadmill and made to run for 60 min at a speed of 25 m/min.

#### 2.2.3 Concurrent training

We evaluated the order of concurrent training. The animals were randomly assigned to either the endurance exercise before resistance exercise group (EE‐RE group) or endurance exercise after the resistance exercise group (RE‐EE group). A 1‐hr rest period was provided between exercises. The animals were trained thrice a week for 3 weeks. (18 exercise sessions in total). In addition, the non‐exercise control (CON) group eliminates the effects of the environment without electrical stimulation or running on a treadmill (CON group anesthetized nine times in total). CON group animals were euthanized in a basal state.

### 2.3 Western blotting

Isolated gastrocnemius muscles were frozen immediately in liquid nitrogen and total muscle protein was extracted by lysis buffer containing 50 mM of HEPES (pH: 7.6), 150 mM of NaCl, 10 mM of EDTA, 10 mM of Na4P2O7, 10 mM of NaF, 2 mM of Na_3_VO_4_, 1% (vol/vol) of NP‐40, 1% (vol/vol) of Na‐deoxycholate, 0.2% (wt/vol) of SDS, and 1% (vol/vol) of a complete protease inhibitor cocktail. The protein concentrations were measured using a Protein Assay Bicinchoninate Kit (Nacalai Tesque Inc.). Just before SDS‐PAGE, an aliquot of the extracted protein solution was mixed with an equal volume of sample loading buffer containing 1% (vol/vol) of 2‐mercaptoethanol, 4% (wt/vol) of SDS, 125 mM of Tris–HCl (pH: 6.8), 10% (wt/vol) of sucrose, and 0.01% (wt/vol) of bromophenol blue; the mixture was then heated at 97°C for 3 min. Ten micrograms of protein were next separated on an SDS‐polyacrylamide gel and electrically transferred from the gel to an Immuno‐Blot PVDF membrane (Bio‐Rad Laboratories). The blot was blocked by Blocking One (Nacalai Tesque) for 1 hr at room temperature and incubated with primary antibodies overnight at 4℃ in TBS with 0.1% Tween‐20. The signals were detected using the ImmunoStar Zeta or LD (Wako Chemicals), quantified by C‐Digit (LI‐COR Biosciences), and expressed as arbitrary units. Coomassie Brilliant Blue (CBB) staining was used to verify consistent loading.

### 2.4 Primary antibodies for western blotting

The following primary antibodies were used for western blotting in the present research: Akt (9272; Cell Signaling Technology), p‐Akt (#4060S; Cell Signaling Technology), mTOR (#2983; Cell Signaling Technology), p‐mTOR (#2971; Cell Signaling Technology), TSC2 (#4308; Cell Signaling Technology), p‐TSC2 (#40729; Cell Signaling Technology), p70S6K (#9202; Cell Signaling Technology), p‐p70S6K (#9205; Cell Signaling Technology), 4E‐binding protein 1 (4E‐BP1) (#9452; Cell Signaling Technology), p‐4E‐BP1 (#9459; Cell Signaling Technology), S6 (#2217; Cell Signaling Technology), p‐S6 (#4858S; Cell Signaling Technology), eukaryotic initiation factor 4B (eIF‐4B) (#3592; Cell Signaling Technology), p‐eIF‐4B (#5399; Cell Signaling Technology), glycogen synthase kinase 3β (GSK‐3β) (#9315; Cell Signaling Technology), p‐GSK‐3β (#9336; Cell Signaling Technology), extracellular signal‐regulated kinase1/2 (Erk1/2) (#9102; Cell Signaling Technology), p‐Erk1/2 (#9101; Cell Signaling Technology), p38 mitogen‐activated protein kinase (p38) (#9212; Cell Signaling Technology) p‐p38 (#9211; Cell Signaling Technology), oxidative phosphorylation (OXPHOS) (ab110413; Abcam), GAPDH (ab8245; Abcam), Cytochrome C (556433; BD Biosciences), AMPK (#2532; Cell Signaling Technology), p‐AMPK (#2531; Cell Signaling Technology), PGC‐1α (516557; Millipore), acetyl‐CoA carboxylase (ACC) (#3676; Cell Signaling Technology), and p‐ACC (#3661; Cell Signaling Technology).

### 2.5 RNA isolation and real‐time polymerase chain reaction (PCR)

Total RNA (mRNA) was isolated from frozen whole gastrocnemius muscles using the TRIzol reagent (Invitrogen). The quantity and quality of RNA were validated with NanoDrop (Thermo Fisher Scientific). Complementary DNA was synthesized using the PrimeScript RT Master Mix (Takara Bio, Inc.). qRT‐PCR was performed with the Thermal Cycler Dice Real‐Time System using SYBR Premix Ex Taq II (Takara Bio, Inc.). The PCR protocol was as follows: denaturation for 15 s at 95°C, then annealing and extension for 40 s at 60°C (40 cycles). The dissociation curve for each sample was analyzed to verify the specificity of each reaction. The relative mRNA expression levels of the target genes were determined by the delta‐delta Ct method and normalized to the expression of TATA‐box binding protein (Tbp). The primer sequences are shown in Table 1.

**TABLE 1:** Primer sequence for RT‐PCR

Gene	Forward primer (5′‐3′)	Reverse primer (5′‐3′)
*Cs*	GCATGAAGGGACTTGTGTA	TCTGGCACTCAGGGATACT
*Cox4*	CTCCAACGAATGGAAGACAG	TGACAACCTTCTTAGGGAAC
*Tfam*	GAAGGGAATGGGAAAGGTAGA	AACAGGACATGGAAAGCAGAT
*Tbp*	CTGCCACACCAGCTTCTGA	TGCAGCAAATCGCTTGGG

Abbreviations: *Cox4,* Cytochrome C oxidase subunit IV; CS, citrate synthase; Tbp, TATAbox binding protein; *Tfam,* mitodhondrial transcription factor A.

### 2.6 Statistical analysis

Data are shown as means ± *SE* of the means. For all measurements, one‐way analyses of variance were conducted. When a significant *p* value was obtained, statistical significance was calculated according to the Tukey's method. The GraphPad Prism 7 software program (GraphPad, Inc.) was used for all statistical calculations and the significance level was set to *p* < .05 for all cases.

## 3 RESULTS

### 3.1 Body and muscle weight

**FIGURE 1:**
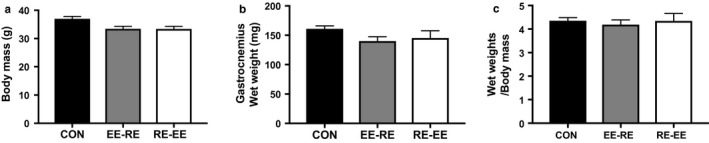
Effects of the order of concurrent training on body mass and gastrocnemius muscle wet weight after 3 weeks of training. Body mass (a), gastrocnemius muscle wet weight (b), and gastrocnemius wet weight per body mass (c) were measured after 3 weeks of concurrent training. Significant differences were not observed between the CON group (*n* = 5), EE‐RE group (*n* = 5), and RE‐EE group (*n* = 5), although statistical analyses of the differences in the training order between the groups were performed

We measured the body mass and gastrocnemius muscle wet weight to confirm the effects of concurrent training and found that 3 weeks of concurrent training did not affect body composition. Further, body mass, gastrocnemius muscle wet weight, and gastrocnemius/body mass were not changed during 3 weeks of concurrent training (Figure 1).

### 3.2 mTOR signaling pathway

**FIGURE 2:**
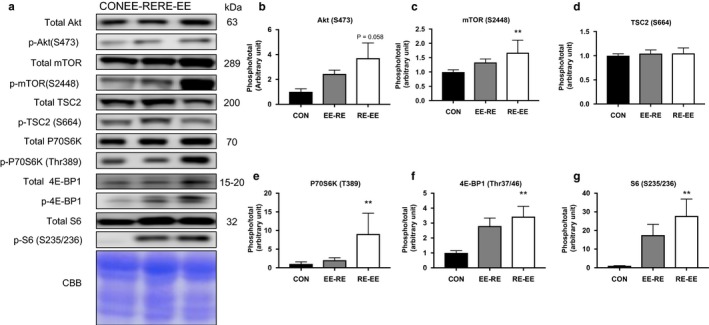
Effects of the order of concurrent training on the mTOR signaling protein in the gastrocnemius muscle. Representative immunoblots are shown in (a). Phosphorylation and protein expression levels of (b) Akt, (c) mTOR, (d) TSC2, (e) P70S6K, (f) 4E‐BP1, and (g) S6 in the gastrocnemius muscle after 3 weeks of concurrent training were analyzed by western blotting. Values represent means ± *SE*. **p < .05 versus the CON group

Separately, concurrent training increased the phosphorylation levels of mTOR signals. We measured the phosphorylation levels of mTOR signaling proteins to investigate the effects of the concurrent training regimen. In this study, we applied electrical stimulation to simulate resistance exercise and designated treadmill running as the endurance exercise. After 3 weeks of training, there was no significant increase observed in Akt, mTOR, or TSC2 (Figure 2b–d), while the phosphorylation levels of P70S6K, 4E‐BP1, and S6 were increased significantly in the RE‐EE group as compared with in the CON group (Figure 2e–g). However, any observed differences were not statistically significant between the EE‐RE group and RE‐EE group.

### 3.3 Other signaling proteins for hypertrophy

**FIGURE 3:**
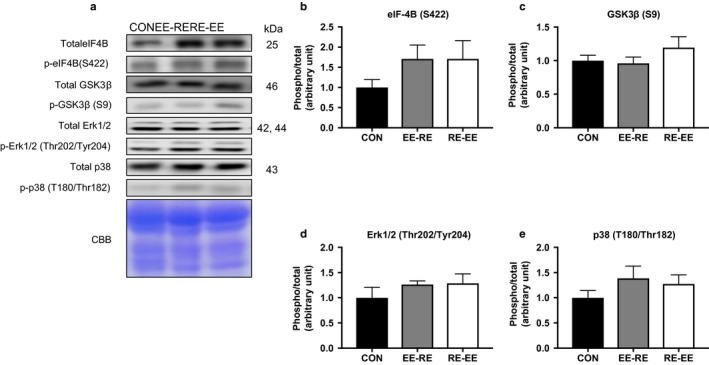
Effects of the order of concurrent training on the signaling protein in the gastrocnemius muscle. Representative immunoblots are shown in (a). Phosphorylation and protein expression levels of (b) eIF‐4B, (c) GSK3B, (d) Erk1/2, and (e) p38 in the gastrocnemius muscle after 3 weeks of concurrent training were analyzed by western blotting. Values represent means ± *SE*

We measured other signaling molecules to quantify muscle hypertrophy. There was no significant differential effect of concurrent training among the groups with regard to other signaling molecules for hypertrophy (Figure 3).

### 3.4 Mitochondrial proteins

**FIGURE 4:**
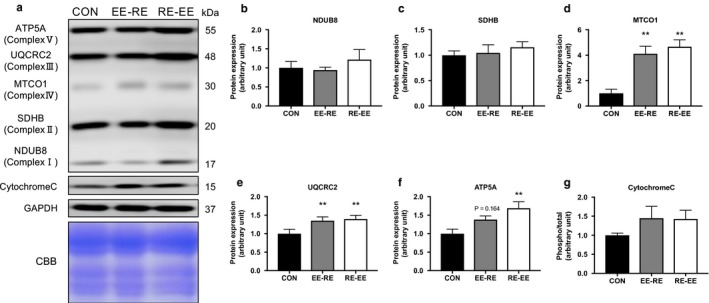
Effects of the order of concurrent training on the mitochondrial proteins in the gastrocnemius muscle. Representative immunoblots are shown in (a). Protein expression levels of (b) NDUB8, (c) SDHB, (d) MTCO1, (e) UQCRC, (f) ATP5A, and (g) Cytochrome C in the gastrocnemius muscle after 3 weeks of concurrent training were analyzed by western blotting. Values represent means ± *SE*. **p < .05 versus the CON group

We measured the expression levels of proteins related to the mitochondrial complex to examine a difference related to the order of concurrent training. After 3 weeks of training, the protein levels of MTCO1 and UQCRC2 were significantly increased in the concurrent training groups as compared with in the CON group, while no differences associated with training order were observed (Figure 4d). Further, there were no significant changes in the protein expression levels of other mitochondrial proteins or cytochrome C (Figure 4b, c, e, and h).

### 3.5 Oxidative metabolism‐related proteins and genes

**FIGURE 5:**
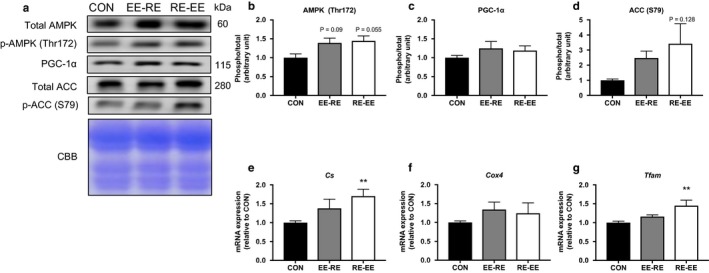
Effects of the order of concurrent training on the protein of endurance capacity and mitochondrial genes in the gastrocnemius muscle. Representative immunoblots are shown in (a). Protein expression levels of (b) AMPK, (c) PGC‐1α, (d) ACC, mRNA expression of (e) Cs, (f) Cox4, and (g) Tfam in the gastrocnemius muscle after 3 weeks of concurrent training were analyzed by western blotting or RT‐PCR. Values represent means ± *SE*. **p < .05 versus the CON group

Differences in the expression levels of proteins related to the oxidative metabolism were not observed between each group (Figure 5b–d). The expression levels of Cs and Tfam significantly increased the RE‐EE group compared with the CON group (Figure 5e and f). Three weeks of concurrent training affect the expression of proteins or mRNA related to the oxidative metabolism (Figure 5).

## 4 DISCUSSION

Previous study has suggested that concurrent training with resistance and endurance training better suppressed muscle hypertrophy in comparison with resistance training alone (Hickson, 1980; Kraemer et al., 1995). Moreover, we hypothesized that the effects of resistance and endurance training can be effectively obtained by changing the order of concurrent training.

First, we examined the existence of effects of the order of concurrent training on body mass and gastrocnemius muscle wet weight and found that the order of concurrent training did not affect either. Concurrent training protocols applied in this study are thought to be able to induce the activation of mTOR signaling. Previous similar study demonstrated that exercise‐training during 3 weeks has significantly altered the molecular signaling (Takahashi, Matsunaga, Hatta, & Kitaoka, 2019). Short duration (21 days) resistance training was increased mTOR signaling (Takegaki, Sase, & Fujita, 2019). In addition, the recent study demonstrated that 7 days of chronic muscle contractile activity (10 Hz, 3 hr/day), an endurance exercise model in rats, increased the protein levels of Mfn2 and Opa1 (Iqybal, Ostojic, Singh, Joseph, & Hood, 2013). In the human study, 2 weeks of high‐intensity interval training was shown to increase protein levels concerning mitochondrial dynamics (Perry, Heigenhauser, Bonen, & Spriet, 2008). Previous studies have shown that the activation of mTOR signaling and mitochondrial adaptation occurs in a shorter period than that applied in this experiment. Our study was performed twice a day (both endurance and resistance exercise) and trained thrice a week for 3 weeks (18 exercise sessions in total); therefore, we think that the experiment protocols are sufficient. Of note, in this study, the experimental period was not long enough for a significant change in weight to feasibly occur, so a longer experimental period may induce muscle weight alterations according to the molecular signal activation evoked by a difference in the order of training.

Next, we reviewed changes in the activation levels of signal molecules for muscle hypertrophy according to the order of concurrent training. Using the mTOR signaling pathway, we found that the RE‐EE group had increased the phosphorylation levels of P70S6K, 4E‐BP1, and S6. Further, the phosphorylation of Akt was increased by either concurrent training order and there was no significant difference between the two. Previous human research has reported that endurance training increases total Akt (Kazior et al., 2016). Also, in animal models, a difference in Akt phosphorylation based on the order of concurrent training was not demonstrated (Ogasawara et al., 2014). Akt is negatively regulated by AMPK via TSC2 phosphorylation (Atherton et al., 2005), but, in this study, TSC2 phosphorylation was not affected by AMPK. The characteristic activation pattern of AMPK involves an increase in such occurring immediately after exercise with a return to the basal level at 1 hr after exercise (Atherton et al., 2005). In this study, gastrocnemius was sampled at 24 hr after the end of the experiment; therefore, AMPK phosphorylation could not be accurately demonstrated. Downstream of the mTOR signaling pathway, P70S6K and S6 were confirmed to significantly increase in the RE‐EE group. In this experiment, the interval between the resistance and endurance exercises, respectively, was 1 hr. Since the response of AMPK returned to the basal level in 1 hr, the AMPK phosphorylation level was not affected in the EE‐RE group and only the effects of the resistance exercise regimen remained. Moreover, we found that the RE‐EE group promoted the downstream effectors of muscle hypertrophy. Previous studies have reported that endurance training improves the effectiveness of resistance training (Kazior et al., 2016). Based on this experiment, we also postulate that endurance training performed later does not negatively control the mTOR signal.

AMPK plays an important role in upregulating oxidative metabolism and gene expression related to mitochondrial function (Fan & Evans, 2017). In this study, the effects of the difference in the order of training on these protein expressions were not observed. As described above, AMPK returned to the basal level at 1 hr after exercise (Atherton et al., 2005). However, in this study, an increasing trend was observed at 24 hr after the end of the experiment. This is considered to be an indicator that the actual basal level of phosphorylation was raised via the 3‐week training period. AMPK is known to activate PGC‐1α, which is the master gene for oxidative and metabolic ability (Atherton et al., 2005). When the protein of the mitochondrial respiratory chain complex was examined, a significant increase was confirmed in MTCO1, UQCRC2, and ATP5A although no changes in the other complexes attributed to the order of training were observed. These results suggest that endurance exercise activates AMPK, PGC‐1α, and mitochondria biogenesis. The gene expression related to mitochondria showed a tendency to increase in conjunction with either order of concurrent training, but there was no significant difference attributed to the order of training. Genes related to mitochondria are known to be altered by PGC‐1α. TFAM is a mitochondrial transcriptional factor that regulates its quantity and activation by the way of PGC‐1α (Theilen, Kunkel, & Tyagi, 2017). In this study, TFAM showed a tendency to increase most significantly in the RE‐EE group, suggesting that the amount of mitochondria varies based on the order of training. Previous studies have reported that the enzymatic activity of the oxidative metabolism did not change after resistance training (Tesch, Thorsson, & Colliander, 1990). Our results also suggested that resistance exercise did not negatively affect genes related to mitochondria. In addition, concurrent training has been found to increase mitochondrial respiratory chain complex proteins. More specifically, resistance training increases respiratory chain complex proteins (Kitaoka, Nakazato, & Ogasawara, 2016), while mitochondrial respiratory function increases in human skeletal muscle after resistance training (Porter, Reidy, Bhattarai, Sidossis, & Rasmussen, 2015). In this study, we obtained similar results to those from previous studies, and the expression level of each gene was increased by either order of concurrent training. However, the two orders did not affect the mitochondrial respiratory chain complex differently.

In conclusion, our results demonstrated that concurrent training could have effects while leading with either resistance training or endurance training. We observed a difference depending on the order of concurrent training on the mTOR signaling pathway. When conducting concurrent training on the same day, the order “RE‐EE” seems to have a relatively noticeable effect on hypertrophy signal, but a change in the order did not impact oxidative metabolism and mitochondrial biogenesis. In future research, the adaptation to skeletal muscle is considered to change depending on the time, frequency, and intensity of each training.

## CONFLICT OF INTEREST

None declared.

## AUTHOR CONTRIBUTIONS

All authors conceived and designed the project; T.S. performed the experiments; T.S. and T.T. analyzed the data; T.S. wrote the paper; T.S. and T.T. made manuscript revisions. All authors read and approved the final manuscript.
